# 

**Published:** 1883-02

**Authors:** 


### CONTENTS.

### COMMUNICATIONS.

On the Relations which Dental Caries, Etc. Continued from last issue ........ 51

### SELECTIONS.

Antiseptics ........ 83
Faith an Element of Success in Practice ........ 85
The Physiology of Secretion ........ 86
The Value of Antiseptics ........ 87
Doctors of Medical Science ........ 88
A Case of Gangrena Oris ........ 89
Decaigne on the Effects of Tobacco ........ 91
Treatment of Scurvy ........ 92
Female Doctors ........ 92

### CORRESPONDENCE.

Researches on the Epithelium of the Cornea ........ 93
In Memoriam ........ 94
A Centenarian ........ 96
Competitive Examination in China ........ 96

### EDITORIAL.

Dental Department of the University of California ........ 97
The Mississippi Valley Dental Association ........ 98
History of the Indiana State Dental Association—1858 to 1882 ........ 99
Ohio Dental College Association ........ 99
Transactions ........ 99
Wants ........ 100

